# Association Between Type 2 Diabetes Mellitus, HbA1c and the Risk for Spontaneous Bacterial Peritonitis in Patients with Decompensated Liver Cirrhosis and Ascites

**DOI:** 10.1038/s41424-018-0053-0

**Published:** 2018-09-24

**Authors:** Tammo L. Tergast, Hans Laser, Svetlana Gerbel, Michael. P. Manns, Markus Cornberg, Benjamin Maasoumy

**Affiliations:** 1 Department of Gastroenterology, Hepatology and Endocrinology, Hannover Medical School, Carl-Neuberg-Str.1, 30625 Hannover, Germany; 2 Centre for Information Management (ZIMt), Hannover Medical School, Carl-Neuberg-Str.1, 30625 Hannover, Germany; 3 German Centre for Infection Research (Deutsches Zentrum für Infektionsforschung DZIF), Partner-site Hannover-Braunschweig, Hannover, Germany; 4 Centre for Individualised Infection Medicine (CIIM), c/o CRC Hannover, Feodor-Lynen-Str. 15, 30625 Hannover, Germany

## Abstract

**Introduction:**

Type 2 diabetes mellitus (DM) is a frequent comorbidity among patients with liver cirrhosis. However, data regarding the impact of DM on spontaneous bacterial peritonitis (SBP) are quite limited. Our aim was to analyze the impact of DM and HbA1c values on the incidence of SBP and mortality in patients with liver cirrhosis and ascites.

**Methods:**

A number of 475 consecutive patients with liver cirrhosis and ascites were analyzed. Presence of DM as well as HbA1c was assessed at the time of the first paracentesis. Patients were followed up for a mean of 266 days. Primary endpoints were SBP development and mortality.

**Results:**

Overall, 118 (25%) patients were diagnosed with DM. DM patients had an increased risk for developing a SBP during follow-up (HR: 1.51; *p* = 0.03). SBP incidence was particularly high in DM patients with HbA1c values ≥6.4%, significantly higher than in DM patients with HbA1c values <6.4% (HR: 4.21; *p* = 0.0002). Of note, DM patients with HbA1c <6.4% at baseline had a similar risk for SBP as those without DM (HR: 0.93; *p* = 0.78, respectively). After excluding all patients who were eligible for secondary antibiotic prophylaxis, HbA1c ≥6.4% but neither bilirubin nor ascites protein level were associated with primary SBP development in the multivariate analysis (*p* = 0.003).

**Conclusions:**

Individuals with liver cirrhosis and concomitant DM have a higher risk for developing a SBP. HbA1c values may be useful to further stratify the risk for SBP among DM patients, which may help to identify those who benefit from antibiotic prophylaxis.

## Introduction

Decompensated liver cirrhosis is associated with significant alterations of various parts of the human immune system leading to a syndrome that is called cirrhosis-associated immune dysfunction. As a result, patients with liver cirrhosis have a higher susceptibility for infections but at the same time may show a hyperinflammatory response after an infection has been acquired^1,2^. Infections often act as a trigger for hepatic decompensation or secondary organ failure like encephalopathy (HE) or acute kidney injury (AKI)^3^ and have been identified as the most important cause of an acute-on-chronic liver failure (ACLF) in Europe^4^. Spontaneous bacterial peritonitis (SBP) is the most frequent type of infection in individuals with decompensated liver cirrhosis^5^. Over the recent years the optimal management of SBP has been intensively discussed. Unfortunately, antibiotic treatment is becoming more and more challenging due to the emergence of multidrug-resistant bacteria, in particular in nosocomial-acquired SBP (nSBP)^3,6^. However, even if an adequate antibiotic regimen is timely initiated many patients will experience complications like AKI or even a fatal outcome^3^. For an optimal management of cirrhotic patients it is important to identify and further validate risk factors for SBP development and for a particularly severe course of SBP. While the impact of co-medication (i.e., proton pump inhibitors) has gained attention in the recent years^7^, the role of comorbidities has widely been neglected in the context of SBP. However, patients with liver cirrhosis often suffer from multiple comorbidities^8^. One of the most common comorbidities is type 2 diabetes mellitus (DM) affecting 20–40% of cirrhotic patients^9–11^. DM is well known to increase the risk for bacterial infections in the general population^12,13^. In patients with liver cirrhosis DM has been associated with a higher risk for hepatic decompensation, encephalopathy and a higher overall mortality^14,15^. However, data on the impact of DM on the risk for SBP are rare. Furthermore, the impact of the severity of blood glucose disturbance in these patients has been completely neglected, so far. Assessment of the level of glycosylated hemoglobin A1c (HbA1c) is a widely used standard diagnostic procedure in the management of DM patients. Currently, HbA1c is widely used as gold standard for DM diagnosis and monitoring of antidiabetic treatment^16^. HbA1c reflects the mean blood sugar level during the last 90–120 days and may therefore be considered as the most appropriate long-time marker for the severity of glucose metabolism dysregulation.

In this study, we aimed to investigate the impact of DM on the hazard for SBP and all-cause mortality in patients with decompensated liver cirrhosis. A particular focus of this study was to analyze the potential value of HbA1c in further stratifying the risk of cirrhotic patients for SBP development and mortality.

## Patients and methods

### Study cohort and inclusion/exclusion criteria

Patients were recruited from the Hannover ascites cohort (HAC), which currently includes >600 patients with decompensated liver cirrhosis and ascites. For the Hannover ascites cohort all consecutive patients with decompensated liver cirrhosis and ascites who were hospitalized between January 2012 and June 2016 and underwent a paracentesis at Hannover Medical School were considered. In order to minimize a potential selection bias patients were automatically identified using the Enterprise Clinical Data Warehouse, which contains data of over 2 million patients and more than 500 million additional data points for clinical information: We conducted an automated research using ICD and laboratory codes relevant for liver diseases and ascites. Afterwards the automatic identification was validated manually using the patients’ medical records. All individuals with either no manifest liver cirrhosis, presence of a secondary intraabdominal infection, stem cell transplantation, history of solid organ transplantation (except for liver transplantation), evidence of a malignancy (except for hepatocellular carcinoma within the MILAN criteria), HIV-infection or congenital immune dysfunction were excluded. For the primary scope of the current study all patients who already had evidence for SBP in the screening paracentesis were withdrawn from analysis. Overall, 475 patients met the inclusion criteria and were further followed up (Supplementary Figure 1). However, follow-up data of patients with SBP at screening paracentesis were used to study the impact of DM on recurrent SBP. For this purpose only patients with SBP at the first paracentesis (*n* = 151), as well as those with a history of SBP (*n* = 70) were considered (Supplementary Figure 2)

### Data collection

Data regarding the history of SBP, presence of esophageal varices and history of variceal bleeding were assessed from the patients’ medical record. DM diagnosis was performed according to the German guidelines^17^ and/or based on the patients’ files. In addition to the epidemiological data, we assessed laboratory values at the time of the first paracentesis. SBP was diagnosed based on a polymorphonuclear leukocyte (PMN) count ≥250 cells/mm^3^ or a total nucleus containing cell count ≥500 cells/mm^3^ in concordance with the German national guideline^18^.

### Study design

In order to determine the relevance of DM a longitudinal study was performed. Two different endpoints were analyzed:

1. Incidence of SBP.
2. All-cause mortality.

One aim of the study was to analyze whether the severity of the disturbance of glucose metabolism was associated with the selected clinical endpoints. Therefore, HbA1c levels at baseline were compared between patients with and without SBP development as well as between those with and without death within 90 days, respectively. Optimal HbA1c cutoff values were identified by using receiver operating characteristic (ROC) curves. All analysis regarding the prognostic value of HbA1c levels were limited to a 90 day follow-up due to the expected increasing variability of HbA1c values during longer observation periods.

### Statistics

Statistical analyses were performed with SPSS (Version 22.0; IBM, New York, USA), GraphPad Prism (version 5.0; GraphPad Software Inc. La Jolla, California, USA) and Microsoft Excel (Microsoft, Redmond, Washington, USA). Continuous variables were calculated with an unpaired *t*-test and are presented as mean with standard deviation. Categorical variables were analyzed with Pearsons’s *χ*^2^-test and are listed as proportions. Kaplan Meyer curves were used to visualize survival curves. Survival curves were calculated using the log-rank test. Furthermore, a univariate Cox-regression was performed including all assessed baseline parameters to determine risk factors for the respective study endpoints. To adjust for potential confounding risk factors all parameters with *p*-values <0.05 in the univariate Cox regression were included in the multivariate Cox-regression analysis using backwards stepwise regression.

### Ethics

This study followed the declaration of Helsinki and was approved by the local ethics committee.

## Results

### Association between DM and the incidence of SBP

A number of 118 (25%) patients of the study cohort were diagnosed with DM. There were a few significant differences in the assessed baseline characteristics between patients with and without DM. Male gender and NASH as an underlying cause of the cirrhosis were more frequent in the DM cohort (67% vs. 56%; *p* = 0.04 and 12% vs. 4%; *p* = 0.002, respectively). Furthermore, individuals with DM were older (58 years vs. 54 years; *p* = 0.002), had a lower leukocyte (6.78/µl vs. 9.28/µl; *p* < 0.0001) and platelet count (117,000/µl vs. 143,000/µl; *p* = 0.002). Of note, mean creatinine was also higher in DM patients (172 µmol/l vs. 142 µmol/l; *p* = 0.04) (Table 1a).

**Table 1a:** Baseline parameters comparing patients with and without DM at the time of the first paracentesis

	Overall	DM	no-DM	p-value
Patients (*n*, %)	475 (100%)	118 (25%)	357 (75%)	
Age (years)	55.34 ± 11.03	58.06 ± 10.36	54.44 ± 11.13	0.002
Male/female (*n*, %)	279 (59%)/196 (41%)	79 (67%)/39 (33%)	200 (56%)/147 (44%)	0.04
*Aetiology*				
NASH (*n*, %)	28 (6%)	14 (12%)	14 (4%)	0.002
HCV (*n*, %)	72 (15%)	16 (14%)	56 (16%)	0.58
ASH (*n*, %)	256 (54%)	55 (47%)	201 (56%)	0.07
Other (*n*, %)	119 (25%)	33 (28%)	86 (24%)	0.40
BP systolic (mmHg)	110 ± 20	111 ± 20	109 ± 20	0.24
BP diastolic (mmHg)	62 ± 12	62 ± 11	62 ± 12	0.70
Leukocytes (10³/µl)	8.66 ± 6.06	6.78 ± 4.40	9.28 ± 6.41	<0.0001
Platelets (10³/µl)	137 ± 96	117 ± 68	143 ± 103	0.002
Hemoglobin (g/dl)	10.09 ± 1.97	10.22 ± 2.09	10.08 ± 1.89	0.70
INR	1.55 ± 0.42	1.48 ± 0.31	1.57 ± 0.46	0.07
Sodium (mmol/l)	134 ± 9	135 ± 5	134 ± 9	0.30
Creatinine (µmol/l)	150 ± 113	172 ± 141	142 ± 102	0.04
AST (× ULN)	2.75 ± 4.23	2.14 ± 1.88	2.85 ± 4.33	0.10
ALT (× ULN)	1.32 ± 2.95	1.04 ± 1.23	1.42 ± 3.34	0.09
AP (× ULN)	1.51 ± 1.37	1.50 ± 1.00	1.52 ± 1.48	0.89
GGT (× ULN)	3.62 ± 4.00	3.75 ± 4.19	3.57 ± 3.94	0.68
CHE (kU/l)	2.11 ± 1.06	2.26 ± 1.11	2.06 ± 1.04	0.15
CRP (mg/l)	35.15 ± 34.66	40.09 ± 44.18	33.49 ± 30.77	0.14
Bilirubin (µmol/l)	108 ± 150	91 ± 154	113 ± 149	0.18
S-Albumin (g/l)	26 ± 7	26 ± 6	26 ± 7	0.84
Ascites-protein (g/l)	13 ± 9	13 ± 9	12 ± 8	0.61
MELD	19.45 ± 7.75	18.97 ± 7.93	19.61 ± 7.70	0.44
Evidence for esophageal varices(*n*, %)	366 (73%)	92 (78%)	254 (71%)	0.15
History of variceal bleeding (*n*, %)	66 (14%)	16 (14%)	50 (14%)	0.90
History of SBP (*n*, %)	70 (15%)	23 (20%)	47 (13%)	0.09
HCC (*n*, %)	16 (3%)	6 (5%)	10 (3%)	0.23
NSBB (*n*, %)	193 (41%)	56 (48%)	137 (38%)	0.08
PPI (*n*, %)	385 (81%)	100 (85%)	285 (80%)	0.24
Rifaximin (*n*, %)	84 (18%)	24 (20%)	60 (17%)	0.52
Norfloxacin (*n*, %)	6 (1%)	2 (2%)	4 (1%)	0.63
Follow-up (days)	266 ± 372	222 ± 329	281 ± 384	0.13

Unpaired *t*-test for continuous data, *χ*^2^-test for categorical data. Parameters shown in mean with standard deviation

**Fig. 1:**
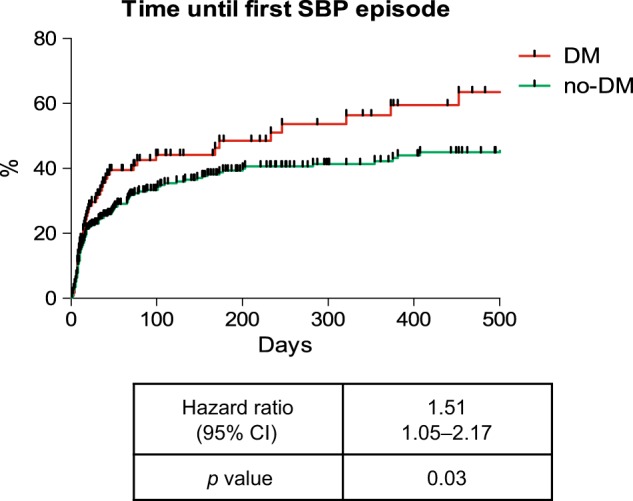
SBP incidence in patients with and without DM. *p*-values were calculated with the log-rank test

Overall, 169 patients experienced at least one SBP episode (Supplementary Table 1). Patients with DM were at an increased risk for developing a SBP during follow-up (HR: 1.51; *p* = 0.03) (Fig. 1). Significant risk factors for SBP development in the univariate analysis included presence of DM, history of SBP, CHE levels and diastolic blood pressure at baseline. However, only DM and CHE remained statistical significant after multivariate analysis (Table 1b).

**Table 1b:** Risk factors for SBP development in the overall cohort

Risk factors for SBP	Univariate			Multivariate		
	HR	95% CI	*p*-value	Adjusted HR	95% CI	*p*-value
Diabetes	1.44	1.03–2.00	0.03	1.51	1.02–2.24	0.04
Diast. BP (mmHg)	0.98	0.97–0.99	0.045	0.99	0.97–1.01	0.26
History of SBP	1.56	1.06–2.27	0.02	1.42	0.88–2.28	0.15
CHE (kU/l)	0.75	0.61–0.92	0.006	0.73	0.60–0.90	0.003

Uni- and multivariate Cox-regression performed with all parameters with p-values <0.05 using backwards stepwise logistic regression.

### Impact of the severity of DM as indicated by HbA1c on the risk for SBP development

**Fig. 2:**
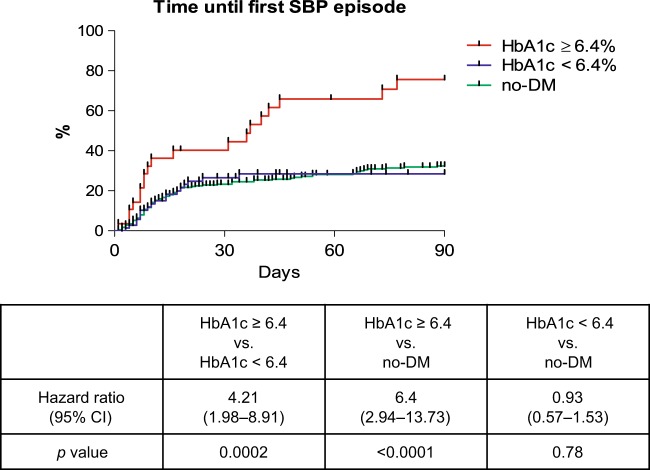
SBP incidence in patients with DM and HbA1c ≥6.4% or <6.4% and no-DM. *p*-values were calculated with the log-rank test

**Fig. 3:**
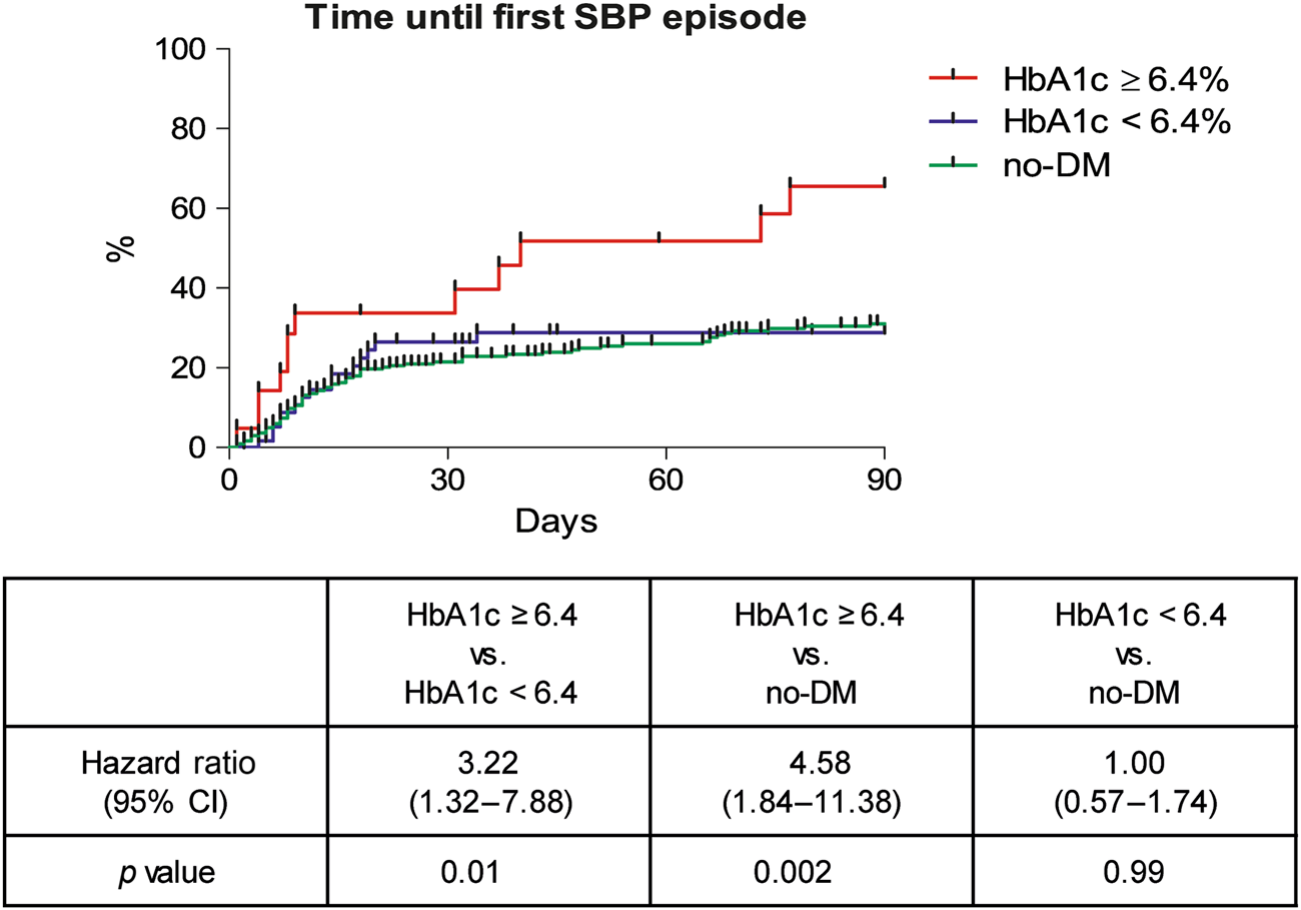
SBP incidence in patients with DM and HbA1c ≥6.4% or <6.4% and no-DM. All patients with history of SBP were excluded in this analysis.*p*-values were calculated with the log-rank test

Data on HbA1c levels were available in 101 out of 118 DM patients (86%). Patients with SBP development within 90 days after baseline had higher HbA1c levels compared to those without SBP (mean HbA1c: 6.4% vs. 5.4%; *p* = 0.002) (Supplementary Table 2). By ROC-curve analysis an HbA1c value of 6.4% was identified as the optimal cutoff to predict a SBP episode within 90 days from baseline (Sensitivity: 84%, Specificity 52%; *p* = 0.009). Patients with HbA1c values ≥6.4% had a significantly higher risk for developing a SBP within 90 days compared to DM patients with values <6.4% (HR: 4.2; *p* = 0.0002) (Fig. 3). Interestingly, DM patients with HbA1c values <6.4% even had a similar SBP incidence as compared to patients without DM (HR: 0.93; *p* = 0.78) (Fig. 2). However, there were a couple of considerable differences in the baseline characteristics between DM patients with an HbA1c ≥6.4% and DM patients with HbA1c values below 6.4%. Patients with an HbA1c ≥6.4% were older (61 years vs. 57 years; *p* = 0.01), had a higher platelet count (133,000/µl vs. 104,000/µl; *p* = 0.047) and more frequently required insulin treatment (93% vs. 64%; *p* = 0.004) (Table 2a, Supplementary Table 4). In order to adjust for these differences uni- and multivariate Cox regression analysis were performed in the DM cohort, including all assessed baseline parameters. Only patients with available HbA1c values were considered. In the univariate analysis an HbA1c-value ≥6.4%, CHE, AP, and leukocytes were identified as potential risk factors for SBP development within 90 days. An HbA1c level ≥6.4%, CHE and AP remained statistical significant risk factors for SBP in the multivariate model (Table 2b). Of note, even in patients with anemia HbA1c values were strongly associated with the development of SBP (Supplementary Figure 3a, b).

**Table 2a:** Baseline characteristics of patients with DM and HbA1c values ≥6.4% or HbA1c values <6.4%

	Overall	HbA1c ≥6.4%	HbA1c <6.4	*p*-value
Patients (*n*, %)	101 (100%)	28 (28%)	73 (72%)	
Age (years)	58.03 ± 9.21	60.91 ± 5.49	56.92 ± 10.16	0.01
Male/female (*n*, %)	63 (63%)/37 (37%)	20 (71%)/8 (29%)	44 (60%)/29 (40%)	0.08
*Aetiology*				
NASH (*n*, %)	13 (13%)	4 (14%)	9 (12%)	0.79
HCV (*n*, %)	14 (14%)	4 (14%)	10 (14%)	0.94
ASH (*n*, %)	45 (45%)	15 (54%)	30 (41%)	0.26
Other (*n*, %)	27 (29%)	5 (18%)	24 (33%)	0.14
BP systolic (mmHg)	112 ± 19	116 ± 21	111 ± 19	0.27
BP diastolic (mmHg)	62 ± 10	65 ± 9	61 ± 11	0.13
Leukocytes (10³/µl)	6.90 ± 4.26	6.50 ± 3.23	7.06 ± 4.64	0.67
Platelets (10³/µl)	112 ± 66	133 ± 72	104 ± 63	0.047
Hemoglobin (g/dl)	10.19 ± 2.06	10.59 ± 2.14	9.98 ± 1.98	0.15
INR	1.49 ± 0.29	1.43 ± 0.23	1.52 ± 0.31	0.21
Sodium (mmol/l)	135 ± 5	136 ± 5	135 ± 6	0.23
Creatinine (µmol/l)	173 ± 145	167 ± 140	176 ± 149	0.77
AST (× ULN)	2.25 ± 1.93	2.17 ± 2.00	2.28 ± 1.94	0.81
ALT (× ULN)	1.08 ± 1.34	1.20 ± 1.61	1.03 ± 1.24	0.59
AP (× ULN)	1.53 ± 1.03	1.53 ± 1.19	1.54 ± 0.97	0.99
GGT (× ULN)	3.94 ± 4.29	4.12 ± 4.29	3.88 ± 4.35	0.81
CHE (kU/l)	2.28 ± 1.10	2.41 ± 1.39	2.22 ± 0.96	0.51
CRP (mg/l)	37.50 ± 40.22	34.25 ± 29.26	38.76 ± 44.12	0.63
Bilirubin (µmol/l)	95 ± 154	88 ± 154	97 ± 155	0.82
S-Albumin (g/l)	26 ± 6	28 ± 6	25 ± 6	0.18
Ascites-protein (g/l)	12 ± 8	14 ± 11	11 ± 5	0.20
MELD	19.27 ± 7.85	17.71 ± 7.81	19.84 ± 7.89	0.23
HbA1c (%)	5.90 ± 1.61	7.81 ± 1.88	5.16 ± 0.58	<0.001
Evidence for esophageal varices (*n*, %)	81 (80%)	23 (82%)	58 (80%)	0.76
History of variceal bleeding (*n*, %)	13 (13%)	4 (14%)	9 (12%)	0.79
History of SBP (*n*, %)	19 (19%)	7 (25%)	12 (16%)	0.32
HCC (*n*, %)	6 (6%)	2 (7%)	4 (6%)	0.75
NSBB (*n*, %)	48 (48%)	17 (61%)	31 (43%)	0.10
PPI (*n*, %)	84 (83%)	23 (82%)	61 (84%)	0.36
Rifaximin (*n*, %)	20 (20%)	4 (14%)	16 (22%)	0.39
Norfloxacin (*n*, %)	1 (1%)	1 (1%)	0 (0%)	0.11

Unpaired *t*-test for continuous data, *χ*^2^-test for categorical data. Parameters shown in mean with standard deviation

**Table 2b:** Risk factors for SBP development in DM patients

Risk factors for SBP	Univariate			Multivariate		
	HR	95% CI	*p*-value	Adjusted HR	95% CI	*p*-value
HbA1c ≥6.4%	3.20	1.68–6.11	<0.0001	4.59	1.98–10.62	<0.0001
CHE (kU/l)	0.52	0.32–0.85	0.009	0.44	0.25–0.78	0.005
AP (× ULN)	1.42	1.05–1.93	0.03	1.72	1.20–2.46	0.003
Leukocytes (10³/µl)	1.06	1.01–1.13	0.04	1.03	0.92–1.14	0.61

Uni- and multivariate Cox-regression performed with all parameters with p-values <0.05 using backwards stepwise logistic regression

### Value of HbA1c in stratifying the risk for SBP in patients eligible for antibiotic prophylaxis

In order to simulate a setting in which primary antibiotic prophylaxis could be considered, all patients with a history of SBP or current norfloxacin treatment were withdrawn from the analysis. A number of 309 no-DM patients and 84 DM patients (*n* = 21 with an HbA1c ≥6.4% and *n* = 63 with an HbA1c <6.4%) remained eligible for the final analysis (Supplementary Table 3). SBP incidence within 90 days was significantly higher in individuals with HbA1c levels ≥6.4% compared to those with levels <6.4% or without DM (HR: 3.22: *p* = 0.01 and HR: 4.58; *p* = 0.002, respectively) (Fig. 3). For the DM patients HbA1c ≥6.4%, platelet count, CHE, and AP were associated with an increased risk for SBP within 90 days in the univariate Cox regression but only HbA1c ≥6.4%, CHE values and AP remained statistically significant in the multivariate approach (Table 3). Of note, in this cohort serum-bilirubin and ascites protein levels were both not significantly associated with the risk for SBP even in the univariate analysis.

**Table 3:** Risk factors for SBP development in DM patients eligble for primary antibiotic prophylaxis

Risk factors for SBP	Univariate			Multivariate		
	HR	95% CI	*p*-value	Adjusted HR	95% CI	*p*-value
HbA1c ≥6.4%	2.61	1.22–5.59	0.01	4.41	1.67–11.60	0.003
Thrombocytes (10³/µl)	1.007	1.001–1.013	0.04	1.004	0.998–1.010	0.17
CHE (kU/l)	0.49	0.27–0.87	0.02	0.38	0.18–0.79	0.01
AP (× ULN)	1.54	1.13–2.11	0.007	1.64	1.14–2.36	0.008

Uni- and multivariate Cox-regression performed with all parameters with p-values <0.05 using backwards stepwise logistic regression excluding all patients with history of SBP

An additional analysis was conducted including all patients that had a SBP in their first paracentesis and with all patients with a documented history of SBP (patients eligible for secondary prophylaxis). DM and HbA1c values ≥6.4% were significantly associated with a higher incidence of recurrent SBP episodes (*p* = 0.04 and *p* = 0.02, respectively) (Supplementary Figures 4a, b).

### Impact of DM on mortality in patients with decompensated liver cirrhosis

**Fig. 4:**
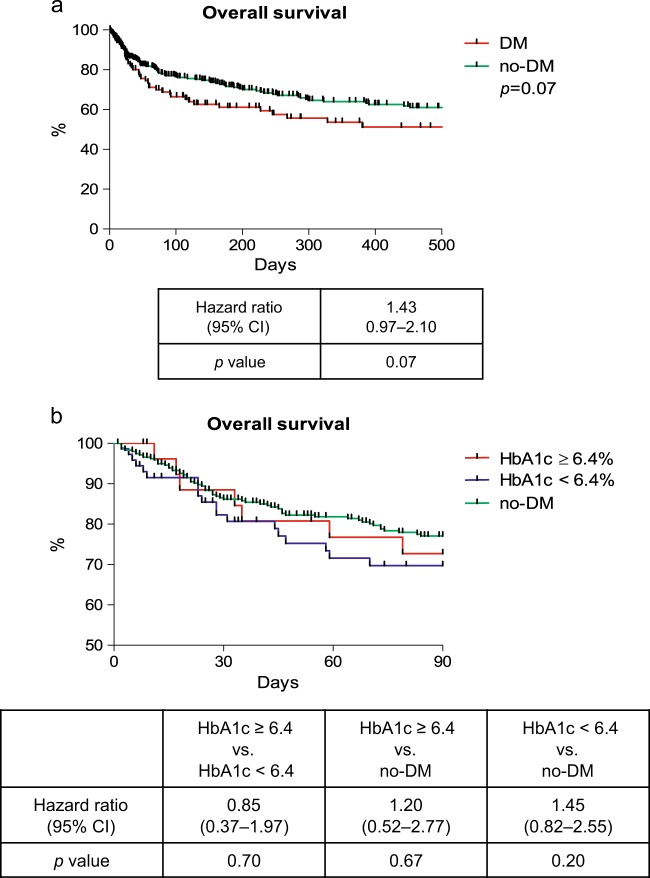
Overall survival in patients with and without DM (**a**) and overall survival in patients with HbA1c values ≥6.4%,  6.4% and no-DM (**b**). *p*-values were calculated with the log-rank test

Overall mortality was numerically higher among DM patients. However, this closely failed to reach statistical significance (HR: 1.43; *p* = 0.07) (Fig. 4a). Similarly, the overall 90-day mortality rate was similar in DM patients with HbA1c levels ≥6.4% and those with values <6.4% (HR: 0.85; *p* = 0.70) (Fig. 4b). Furthermore, neither DM nor HbA1c was identified as significant risk factor for mortality in the uni- and multivariate Cox regression analysis (Supplementary Table 5,6).

### Impact of DM on mortality in patients with SBP

**Fig. 5:**
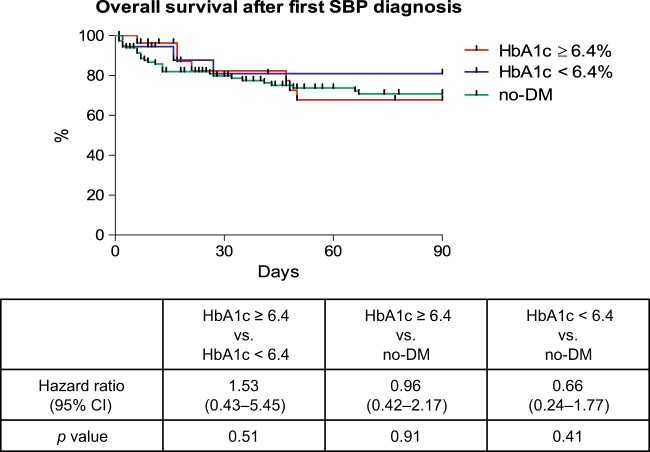
Overall mortality in patients with and without DM after the diagnosis of SBP. *p*-values were calculated with the log-rank test

There was no difference in the detected microorganisms in the ascites cultures between no-DM and DM patients with HbA1c levels < and ≥6.4% (Supplementary Table 7a, b). Estimated mortality within 90-days after SBP diagnosis was neither significantly associated to the presence of DM nor HbA1c values (HR: 1.53; *p* = 0.51 and HR: 0.96; *p* = 0.91, respectively) (Fig. 5).

## Discussion

Development of SBP is a severe complication in patients with liver cirrhosis and often indicates a significant acceleration of the natural history of the disease^19^. Over the recent years several studies tried to identify risk factors for either a higher likelihood of SBP development or a more severe course of SBP. However, the role of comorbidities has rarely been investigated in this context. In this study, we show that the presence of DM leads to significant increase of SBP incidence. Importantly, we were able to demonstrate for the first time that the HbA1c level, an established marker for the severity of blood glucose dysregulation, may serve as a valuable tool to further stratify the risk for SBP in patients with decompensated liver cirrhosis.

Presence of DM is associated with a more complicated course of cirrhosis and a higher likelihood for hepatic decompensation^15,20^. Kwon et al. reported that an insufficient glycemic control is associated with a higher mortality among cirrhotic patients with HCV infection^21^. DM increases the incidence of HCC^22^ and quite a few studies could show that DM is also a relevant risk factor for the development of HE^14,15,20,23,24^. Therefore, it is quite surprising that the impact of DM on the risk for SBP has rarely been studied in the past. In line with our data, Liu et al. documented an increased incidence of SBP in a large US cohort in an analysis based on ICD-9 coded diagnoses^15^.

Similar results have been observed in a smaller cohort from the Netherlands^11^. In a large cohort from France DM increased the overall risk for bacterial infections in patients with cirrhosis, while SBP was not specifically analyzed^20^. There are a couple of possible explanations for the increased SBP incidence among the DM patients. DM leads to significant alterations of the human immune system, including an impaired leukocyte function^13,25^, which adds to the altered immune functions that are caused by liver cirrhosis^2^. Furthermore, DM-induced polyneuropathia may lead to dyskinesia of the gastral and bowels muscles resulting in a prolonged intestinal transit time^26,27^. This may result in an increased risk for bacterial translocation from the gut, which is a key part in SBP pathogenesis^28^.

One of the major findings of our study is that we were able to demonstrate for the first time that the risk for SBP in DM patients is depended on the degree of blood glucose disturbance as indicated by the HbA1c level. While the value of HbA1c as a marker for infections in patients with cirrhosis has not been investigated so far, there are quite some data available demonstrating the link between HbA1c values and DM specific complications like retinopathy and nephropathy^29–31^. Moreover, HbA1c is one of the central markers to adjust antidiabetic treatment^16^. One might argue that the identified HbA1c threshold of 6.4% in our study might be quite low given the fact that values around 6.5–7.5% are currently suggested as target levels to indicate a sufficient treatment^16^. However, it has to be considered that HbA1c target values have not been validated for patients with liver cirrhosis, in particular not for those with decompensated disease and portal hypertension. Portal hypertension may lead to an increased hemolysis and splenomegaly, which both results in a decreased survival time of erythrocytes^32^. This may help to explain why HbA1c values are in general lower in DM patients with compared to those without liver cirrhosis, often even within normal ranges (<6.5%), despite a significant degree blood glucose dysregulation^33–35^.

Given the fact that DM was linked to SBP development in our study and has previously been associated with HE^14,15,20,22–24^ as well as other severe complications of cirrhosis, it seems to be quite likely that it also impairs the overall survival in patients with liver cirrhosis. Indeed, a higher mortality in cirrhotic patients with DM compared to those without DM has been reported from some centers^20,36^. In our study we documented only a numerical higher mortality rate in the DM group. Of note, no difference in the 90-day mortality rate was found between those with HbA1c levels < and ≥ 6.4% despite the significant differences in SBP incidence. However, it has to be considered that patients with lower HbA1c levels in our cohort had a higher MELD score and lower platelets, which may indicate more severe liver disease in this group. Furthermore, it has been shown in non-cirrhotic cohorts that very low HbA1c levels are even associated with a higher overall mortality in DM patients most likely due to a higher risk for hypoglycemic episodes^37^. Therefore, an intensified antidiabetic treatment should not be generally recommended. Interestingly, AP seems to have a predictive value for mortality in our cohort, which might be further investigated in future studies.

Presence of DM leads to significant changes of the gut microbiota^38^. Therefore, it would have been quite convincing if there had been a relevant change in the pathogens that are involved in SBP episodes in DM patients. However, this was not the case in our cohort.

Our study has some important limitations which need to be considered while interpreting the results. Although all patients were included consecutively, the analysis has been performed retrospectively. Therefore, the identified HbA1c threshold needs to be further validated prospectively in a larger setting before specific recommendation can be made for patient management. Furthermore, we unfortunately did not have access to multiple HbA1c values over a longer follow-up period, which would have allowed a more detailed analysis.

However, we are convinced that our results have still some important implications for clinical practice and future studies. Even if an adequate treatment is timely initiated many patients with cirrhosis and SBP will experience severe complications like AKI or a fatal outcome^3^. In the past, prophylactic treatment with antibiotics has been shown to be effective in preventing SBP episodes in selected patients and to improve survival in individuals with a high risk for a severe course of SBP^5,28,39^. However, to achieve a reasonable risk/benefit ratio a wise and specific selection of patients for prophylactic treatments is essential. Currently suggested risk factors for the selection of patients for prophylaxis include an impaired liver function, a decreased ascites protein level, elevated creatinine level and a history of a previous SBP episode^5,28,39^. According to our data, presence of DM and more specifically in case of higher HbA1c levels should be further evaluated as possible indication for antibiotic prophylaxis in patients with cirrhosis to prevent SBP development.

In summary, we demonstrated that DM is an important, independent risk factor for SBP development in cirrhotic patients with ascites. Importantly, we could show for the first time that the risk for SBP in DM patients can be further stratified using HbA1c values as long-time marker for the severity of dysregulation of glucose metabolism.

### Study Highlights

#### What is current knowledge

- Spontaneous bacterial peritonitis (SBP) is the most frequent type of infection in individuals with decompensated liver cirrhosis.
- Type 2 diabetes mellitus (DM) is one of the most common comorbidities in patients with liver cirrhosis affecting 20–40% of cirrhotic patients.

#### What is new here

- DM is associated with a significantly increased risk for the development of a SBP.
- The risk for SBP in DM patients is linked to levels of glycosylated hemoglobin A1c (HbA1c).

## Electronic supplementary material

Supplementary Figure 1

Supplementary Figure 2

Supplementary Figure 3

Supplementary Figure 4

Supplementary Figures legends

Supplementary Tables
