# Supplementary figures and images for: Association Between Type 2 Diabetes Mellitus, HbA1c and the Risk for Spontaneous Bacterial Peritonitis in Patients with Decompensated Liver Cirrhosis and Ascites
Source: Clin Transl Gastroenterol. 2018 Sep 24;9(9):189. doi: 10.1038/s41424-018-0053-0 (PMC6155293; doi:10.1038/s41424-018-0053-0)

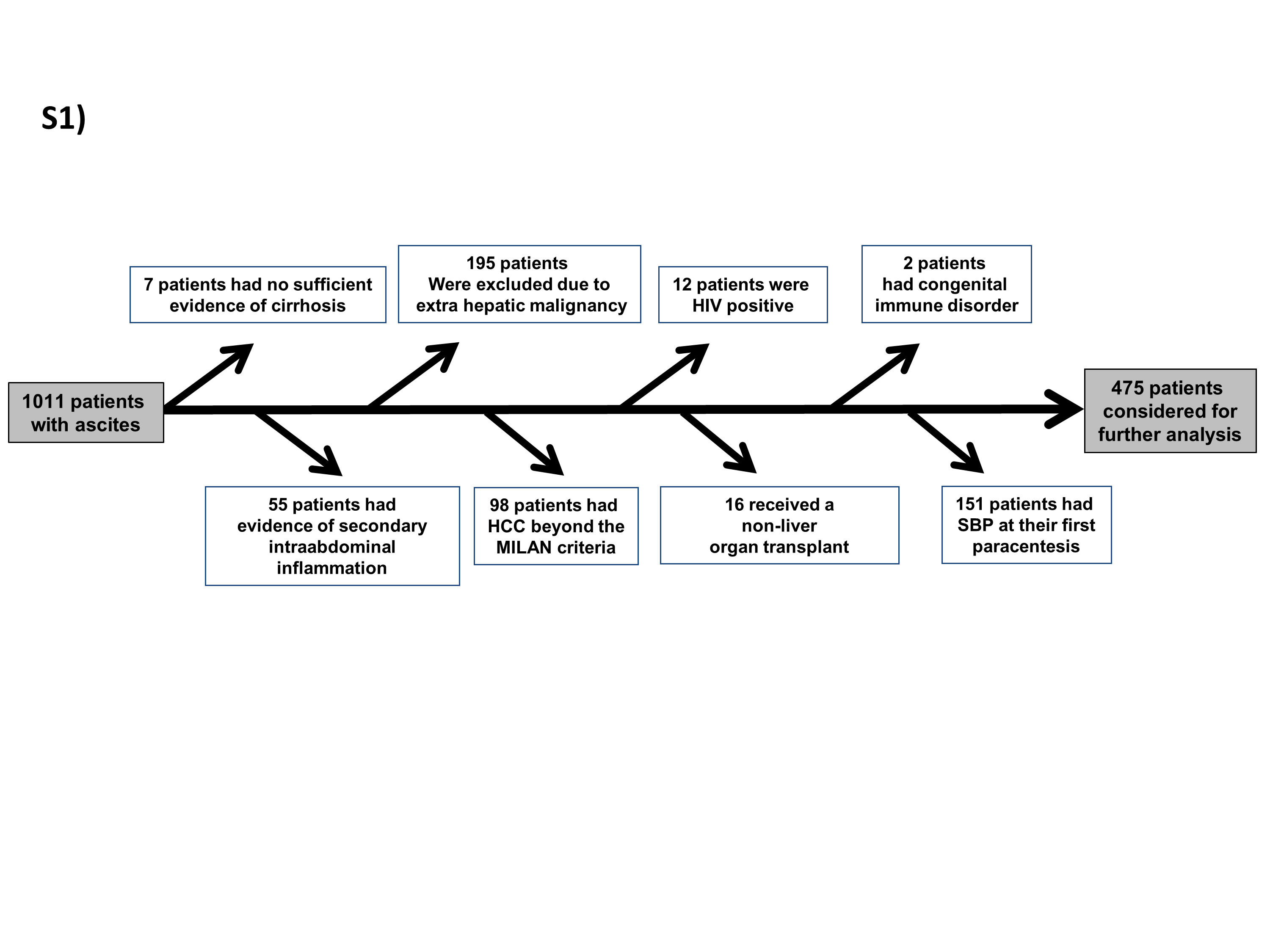

Supplement: Supplementary file 1 — Supplementary Figure 1 [file 41424_2018_53_MOESM1_ESM.tif]

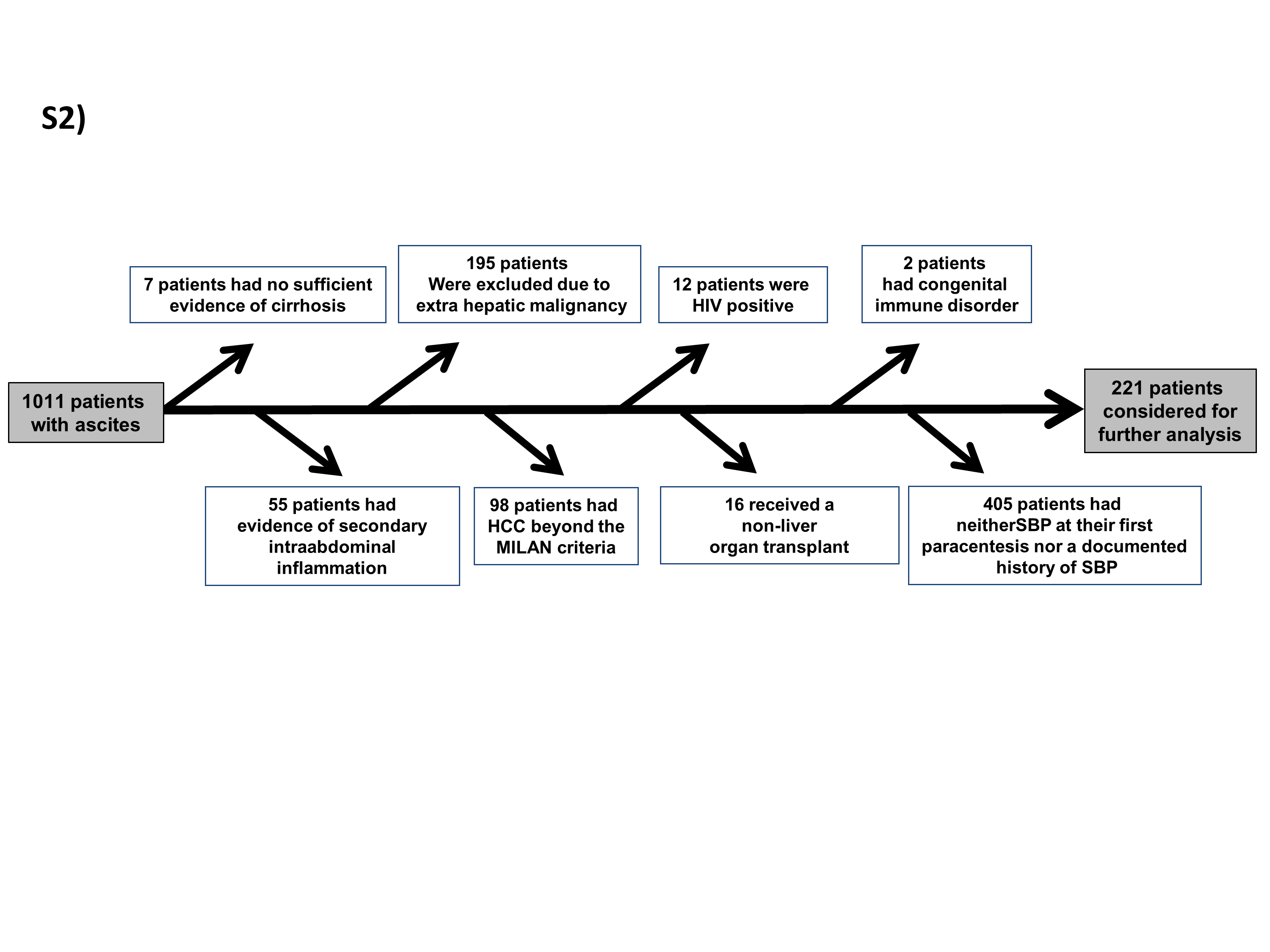

Supplement: Supplementary file 2 — Supplementary Figure 2 [file 41424_2018_53_MOESM2_ESM.tif]
