# Supplementary material for: Association Between Type 2 Diabetes Mellitus, HbA1c and the Risk for Spontaneous Bacterial Peritonitis in Patients with Decompensated Liver Cirrhosis and Ascites
Source: Clin Transl Gastroenterol. 2018 Sep 24;9(9):189. doi: 10.1038/s41424-018-0053-0 (PMC6155293; doi:10.1038/s41424-018-0053-0)
Supplement: Supplementary file 5 — Supplementary Figures legends [file 41424_2018_53_MOESM5_ESM.docx]

**Supplementary Figures:**

**Figure S1:**

Recruitment of the study cohort with applied exclusion criteria.

**Figure S2:**

Recruitment of the study cohort for the analysis shown in supplementary figure 4a.

**Figure S3a:**

SBP incidence in patients with DM and HbA1c values ≥6.4% (n=22) and <6.4% (n=61), only including patients with a Hb <12.5g/dl. P-values were calculated with the log-rank test.

**Figure S3b:**

SBP incidence in patients with DM and HbA1c ≥6.4% (n= 10), <6.4% (n=40), only including patients with a Hb <10g/dl. P-values were calculated with the log-rank test.

**Figure S4a:**

Incidence of recurrent SBP in patients with DM (n=55) and without DM (n=166), only including patients with a SBP at their first paracentesis or with a documented history of SBP. P-values were calculated with the log-rank test.

**Figure S4b:**

Incidence of recurrent SBP in patients with DM and HbA1c values ≥6.4% (n=21) and <6.4% (n=22), only including patients with a SBP at their first paracentesis or with a documented history of SBP. P-values were calculated with the log-rank test.
