# Supplementary material for: Association Between Type 2 Diabetes Mellitus, HbA1c and the Risk for Spontaneous Bacterial Peritonitis in Patients with Decompensated Liver Cirrhosis and Ascites
Source: Clin Transl Gastroenterol. 2018 Sep 24;9(9):189. doi: 10.1038/s41424-018-0053-0 (PMC6155293; doi:10.1038/s41424-018-0053-0)
Supplement: Supplementary file 6 — Supplementary Tables [file 41424_2018_53_MOESM6_ESM.docx]

| Patients with SBP development | Overall | SBP development | No SBP development | p-value |
| --- | --- | --- | --- | --- |
| Patients (n, %) | 475 (100%) | 169 (36%) | 306 (64%) |  |
| Age (years) | 55.34 ± 11.03 | 55.38 ± 10.43 | 55.32 ± 11.35 | 0.96 |
| Male/female (n, %) | 279 (59%) / 196 (41%) | 105 (62%) / 64 (38%) | 174 (57%) / 132 (43%) | 0.26 |
| Aetiology |  |  |  |  |
| NASH (n, %) | 28 (6%) | 11 (7%) | 17 (6%) | 0.67 |
| HCV (n, %) | 72 (15%) | 25 (15%) | 47 (15%) | 0.87 |
| ASH (n, %) | 256 (54%) | 86 (51%) | 170 (56%) | 0.33 |
| Other (n, %) | 119 (25%) | 47 (28%) | 72 (24%) | 0.30 |
| DM | 118 (25%) | 50 (30%) | 68 (22%) | *0.04* |
| BP systolic (mmHg) | 110 ± 20 | 109 ± 19 | 110 ± 21 | 0.42 |
| BP diastolic (mmHg) | 62 ± 12 | 61 ± 11 | 62 ± 13 | 0.40 |
| Leukocytes (10³/µl) | 8.66 ± 6.06 | 8.77 ±6.08 | 8.59 ± 6.06 | 0.76 |
| Platelets (10³/µl) | 137 ± 96 | 145 ± 104 | 133 ± 91 | 0.19 |
| Hemoglobin (g/dl) | 10.09 ± 1.97 | 10.11 ± 1.85 | 10.49 ± 5.45 | 0.56 |
| INR | 1.55 ± 0.42 | 1.54 ± 0.39 | 1.55 ± 0.44 | 0.92 |
| Sodium (mmol/l) | 134 ± 9 | 134 ± 6 | 135 ± 10 | 0.65 |
| Creatinine (µmol/l) | 150 ± 113 | 153 ± 106 | 149 ± 117 | 0.71 |
| AST (x ULN) | 2.75 ± 4.23 | 2.59 ± 4.10 | 2.85 ± 4.30 | 0.54 |
| ALT (x ULN) | 1.32 ± 2.95 | 1.19 ± 2.43 | 1.43 ± 3.25 | 0.42 |
| AP (x ULN) | 1.51 ± 1.37 | 1.54 ± 1.32 | 1.50 ± 1.40 | 0.74 |
| GGT (x ULN) | 3.62 ± 4.00 | 3.64 ± 3.84 | 3.60 ± 4.09 | 0.92 |
| CHE (kU/l) | 2.11 ± 1.06 | 2.01 ± 1.03 | 2.17 ± 1.07 | 0.10 |
| CRP (mg/l) | 35.15 ± 34.66 | 32.67 ± 34.00 | 36.56 ± 34.96 | 0.25 |
| Bilirubin (µmol/l) | 108 ± 150 | 95 ± 140 | 115 ± 155 | 0.16 |
| S-Albumin (g/l) | 26 ± 7 | 25 ± 7 | 26 ± 7 | 0.24 |
| Ascites-protein (g/l) | 13 ± 9 | 14 ± 10 | 12 ± 8 | 0.30 |
| MELD | 19.45 ± 7.75 | 19.38 ± 6.97 | 19.56 ± 8.07 | 0.81 |
| Evidence for esophageal varices (n, %) | 366 (73%) | 131 (78%) | 215 (70%) | 0.09 |
| History of variceal bleeding (n, %) | 66 (14%) | 25 (15%) | 41 (13%) | 0.67 |
| History of SBP (n, %) | 70 (15%) | 33 (20%) | 37 (9%) | *0.03* |
| HCC (n, %) | 16 (3%) | 8 (5%) | 8 (3%) | 0.22 |
| NSBB (n, %) | 193 (41%) | 94 (56%) | 144 (47%) | 0.07 |
| PPI (n, %) | 385 (81%) | 148 (88%) | 237 (78%) | *0.01* |
| Rifaximin (n, %) | 84 (18%) | 32 (19%) | 52 (17%) | 0.60 |
| Norfloxacin (n, %) | 6 (1%) | 3 (2%) | 3 (1%) | 0.46 |
| Follow-up (days) | 266 ± 372 | 295 ± 387 | 245 ± 363 | 0.16 |

**Supplementary Table 1**

**Supplementary Table 2**

| DM Patients | Overall | SBP development | No SBP development | p-value |
| --- | --- | --- | --- | --- |
| Patients (n, %) | 101 (100%) | 37 (37%) | 64 (64%) |  |
| Age (years) | 58.03 ± 9.21 | 59.40 ± 9.12 | 57.24 ± 9.17 | 0.26 |
| Male/female (n, %) | 63 (63%) / 37 (37%) | 25 (68%) / 12 (32%) | 39 (61%) / 25 (39%) | 0.50 |
| Aetiology |  |  |  |  |
| NASH (n, %) | 13 (13%) | 4 (11%) | 9 (14%) | 0.51 |
| HCV (n, %) | 14 (14%) | 4 (11%) | 10 (16%) | 0.64 |
| ASH (n, %) | 45 (45%) | 18 (49%) | 27 (42%) | 0.53 |
| Other (n, %) | 29 (29%) | 11 (30%) | 18 (28%) | 0.99 |
| BP systolic (mmHg) | 112 ± 19 | 113 ± 17 | 112 ± 20 | 0.81 |
| BP diastolic (mmHg) | 62 ± 10 | 62 ± 9 | 63 ± 11 | 0.65 |
| Leukocytes (10³/µl) | 6.90 ± 4.26 | 7.67 ± 4.22 | 6.48 ± 4.19 | 0.19 |
| Platelets (10³/µl) | 112 ± 66 | 127 ± 73 | 104 ± 60 | 0.10 |
| Hemoglobin (g/dl) | 10.19 ± 2.06 | 10.11 ± 1.97 | 10.22 ± 2.10 | 0.81 |
| INR | 1.49 ± 0.29 | 1.54 ± 0.28 | 1.47 ± 0.29 | 0.22 |
| Sodium (mmol/l) | 135 ± 5 | 134 ± 5 | 136 ± 5 | 0.16 |
| Creatinine (µmol/l) | 173 ± 145 | 167 ± 137 | 177 ± 148 | 0.74 |
| AST (x ULN) | 2.25 ± 1.93 | 2.36 ± 2.25 | 2.17 ± 1.70 | 0.65 |
| ALT (x ULN) | 1.08 ± 1.34 | 1.07 ± 1.38 | 1.10 ± 1.31 | 0.91 |
| AP (x ULN) | 1.53 ± 1.03 | 1.78 ± 1.39 | 1.40 ± 0.71 | 0.10 |
| GGT (x ULN) | 3.94 ± 4.29 | 3.70 ± 3.60 | 4.08 ± 4.59 | 0.69 |
| CHE (kU/l) | 2.28 ± 1.10 | 1.88 ± 0.92 | 2.55 ± 1.11 | *0.01* |
| CRP (mg/l) | 37.50 ± 40.22 | 45.01 ± 52.01 | 33.23 ± 30.65 | 0.17 |
| Bilirubin (µmol/l) | 95 ± 154 | 85 ± 130 | 99 ± 164 | 0.66 |
| S-Albumin (g/l) | 26 ± 6 | 26 ± 6 | 26 ± 6 | 0.84 |
| Ascites-protein (g/l) | 12 ± 8 | 15 ± 11 | 11 ± 8 | *0.04* |
| MELD | 19.27 ± 7.85 | 19.56 ± 5.94 | 19.20 ± 8.21 | 0.82 |
| HbA1c (%) | 5.90 ± 1.61 | 6.4 ± 2.1 | 5.4 ± 1.1 | *0.002* |
| Evidence for esophageal varices (n, %) | 81 (80%) | 28 (76%) | 53 (83%) | 0.39 |
| History of variceal bleeding (n, %) | 13 (13%) | 6 (16%) | 7 (11%) | 0.45 |
| History of SBP (n, %) | 19 (19%) | 10 (27%) | 9 (14%) | 0.11 |
| HCC (n, %) | 6 (6%) | 4 (11%) | 2 (3%) | 0.12 |
| NSBB (n, %) | 48 (48%) | 17 (46%) | 31 (48%) | 0.81 |
| PPI (n, %) | 84 (84%) | 34 (92%) | 50 (78%) | 0.08 |
| Rifaximin (n, %) | 20 (20%) | 9 (24%) | 11 (22%) | 0.39 |
| Norfloxacin (n, %) | 1 (1%) | 1 (3%) | 0 (0%) | 0.19 |

**Supplementary Table 3**

|  | Overall | SBP development | No SBP development | p-value |
| --- | --- | --- | --- | --- |
| Patients (n, %) | 393 (100%) | 102 (26%) | 291 (74%) |  |
| Age (years) | 55.28 ± 11.19 | 55.59 ± 9.67 | 55.17 ± 11.67 | 0.74 |
| Male/female (n, %) | 227 (58%) / 166 (42%) | 61 / 41 | 166 / 125 | 0.24 |
| Aetiology |  |  |  |  |
| NASH (n, %) | 17 (4%) | 5 (5%) | 12 (4%) | 0.74 |
| HCV (n, %) | 60 (15%) | 15 (15%) | 45 (16%) | 0.85 |
| ASH (n, %) | 214 (55%) | 56 (56%) | 158 (54%) | 0.92 |
| Other (n, %) | 102 (26%) | 26 (26%) | 76 (26%) | 0.90 |
| DM | 84 (21%) | 30 (29%) | 54 (19%) | *0.02* |
| HbA1c (%) | 5.81 ± 1.28 | 6.31 ± 1.46 | 5.61 ± 1.12 | *0.04* |
| BP systolic (mmHg) | 110 ± 21 | 108 ± 18 | 111 ± 22 | 0.21 |
| BP diastolic (mmHg) | 62 ± 12 | 61 ± 11 | 63 ± 13 | 0.22 |
| Leukocytes (10³/µl) | 8.73 ± 6.12 | 9.76 ± 6.49 | 8.38 ± 5.95 | 0.05 |
| Platelets (10³/µl) | 136 ± 90 | 145 ± 87 | 133 ± 91 | 0.24 |
| Hemoglobin (g/dl) | 10.38 ± 4.49 | 10.20 ± 1.86 | 10.44 ± 5.10 | 0.65 |
| INR | 1.55 ± 0.45 | 1.60 ± 0.45 | 1.53 ± 0.44 | 0.17 |
| Sodium (mmol/l) | 135 ± 6 | 134 ± 6 | 136 ±5 | *0.01* |
| Creatinine (µmol/l) | 148 ± 109 | 169 ± 122 | 140 ± 103 | *0.02* |
| AST (x ULN) | 2.80 ± 3.62 | 3.09 ± 5.18 | 2.71 ± 2.87 | 0.39 |
| ALT (x ULN) | 1.43 ± 3.12 | 1.41 ± 3.09 | 1.44 ± 3.13 | 0.93 |
| AP (x ULN) | 1.55 ± 1.46 | 1.77 ± 1.59 | 1.47 ± 1.40 | 0.10 |
| GGT (x ULN) | 3.70 ± 4.1 | 4.24 ± 4.27 | 3.51 ± 4.02 | 0.14 |
| CHE (kU/l) | 2.18 ± 1.09 | 1.94 ± 0.91 | 2.27 ± 1.14 | *0.03* |
| CRP (mg/l) | 33.86 ± 31.92 | 32.96 ± 31.67 | 34.18 ± 32.12 | 0.71 |
| Bilirubin (µmol/l) | 114 ± 154 | 117 ± 160 | 113 ± 152 | 0.83 |
| S-Albumin (g/l) | 26 ± 7 | 26 ± 8 | 27 ± 7 | 0.33 |
| Ascites-protein (g/l) | 13 ± 8 | 13 ± 8 | 13 ± 8 | 0.98 |
| MELD | 19.60 ± 7.81 | 21.07 ± 6.98 | 19.08 ± 8.02 | *0.03* |
| Evidence for esophageal varices (n, %) | 281 (72%) | 77 (76%) | 204 (70%) | 0.30 |
| History of variceal bleeding (n, %) | 48 (12%) | 11 (11%) | 37 (13%) | 0.59 |
| HCC (n, %) | 15 (4%) | 6 (6%) | 9 (3%) | 0.21 |
| NSBB (n, %) | 192 (49%) | 57 (56%) | 135 (46%) | 0.10 |
| PPI (n, %) | 310 (79%) | 87 (85%) | 223 (77%) | 0.07 |

**Supplementary Table 4**

|  | Overall | HbA1c ≥6.4% | HbA1c <6.4 | p-value |
| --- | --- | --- | --- | --- |
| Patients (n, %) | 101 (100%) | 28 (28%) | 73 (72%) |  |
| Insulin dependency (n, %) | 73 (72%) | 26 (93%) | 47 (64%) | *0.004* |
| Metformin (n, %) | 8 (8%) | 1 (4%) | 7 (10%) | 0.32 |
| Glinides (n, %) | 1 (1%) | 0 (0%) | 1 (1%) | 0.53 |
| Gliptins (n, %) | 7 (7%) | 2 (7%) | 5 (7%) | 0.96 |

**Supplementary Table 5**

| *Risk factors for mortality* | Univariate |  |  |  | Multivariate |  |  |
| --- | --- | --- | --- | --- | --- | --- | --- |
|  | HR | 95% CI | p-value |  | adjusted HR | 95% CI | p-value |
| Diast. BP (mmHg) | 0.98 | 0.97 – 0.98 | 0.03 |  | 0.99 | 0.96 – 1.02 | 0.36 |
| CHE (kU/l) | 0.62 | 0.48 – 0.80 | <0.001 |  | 0.76 | 0.50 – 1.14 | 0.18 |
| SBP development | 1.46 | 1.05 – 2.03 | 0.03 |  | 1.89 | 1.02 – 3.43 | *0.04* |
| MELD | 1.002 | 1.001 – 1.003 | 0.001 |  | 1.07 | 1.04 – 1.10 | *<0.001* |
| AST (xULN) | 1.07 | 1.03 – 1.11 | 0.002 |  | 1.06 | 1.02 – 1.10 | *0.001* |

**Supplementary Table 6**

| *Risk factors for mortality* | Univariate |  |  |  | Univariate |  |  |
| --- | --- | --- | --- | --- | --- | --- | --- |
|  | HR | 95% CI | p-value |  | adjusted HR | 95% CI | p-value |
| Leukocytes (10³/µl) | 1.11 | 1.03 – 1.19 | 0.006 |  | 1.03 | 0.94 – 1.12 | 0.53 |
| MELD | 1.09 | 1.04 – 1.14 | <0.001 |  | 1.09 | 1.04 – 1.15 | <0.001 |
| AP (xULN) | 1.25 | 1.02 – 1.54 | 0.03 |  | 1.50 | 1.03 – 2.17 | 0.04 |

**Supplementary Table 7a**

| Microorgansims | Overall | DM | no-DM | p-value |
| --- | --- | --- | --- | --- |
| Patients (n, %) | 160 (100%) | 50 (31%) | 110 (69%) |  |
| Positive bacterial culture (n, %) | 47 (29%) | 20 (40%) | 27 (25%) | *0.047* |
| Total Gram-negative bacteria (n, %) | 20 (13%) | 9 (18%) | 11 (10%) | 0.10 |
| E. coli (n, %) | 11 (7%) | 5 (10%) | 6 (5%) | 0.25 |
| Klebsiella spp. (n, %) | 6 (4%) | 2 (4%) | 4 (4%) | 0.91 |
| Other Gram-negative bacilli (n, %) | 3 (2%) | 2 (4%) | 1 (1%) | 0.18 |
|  |  |  |  |  |
| Gram-positive bacteria (n, %) | 34 (21%) | 14 (28%) | 20 (18%) | 0.16 |
| Enterococcus spp. (n, %) | 11 (7%) | 4 (8%) | 7 (6%) | 0.71 |
| Staphylococcus spp. (n, %) | 9 (6%) | 3 (6%) | 6 (5%) | 0.89 |
| Streptococcus spp. (n, %) | 9 (6%) | 5 (10%) | 4 (4%) | 0.11 |
| MRSA (n, %) | 3 (2%) | 1 (2%) | 2 (2%) | 0.94 |
| Other Gram-positive bacilli (n, %) | 4 (3%) | 1 (2%) | 3 (3%) | 0.79 |
|  |  |  |  |  |
| Fungi (n, %) | 2 (1%) | 1 (2%) | 1 (1%) | 0.57 |
| Candida spp. (n, %) | 2 (1%) | 1 (2%) | 1 (1%) | 0.57 |

**Supplementary Table 7b**

| Microorgansims | Overall | HbA1c ≥6.4 | HbA1c <6.4 | p-value |
| --- | --- | --- | --- | --- |
| Patients (n, %) | 44 (100%) | 22 (50%) | 22 (50%) |  |
| Positive bacterial culture (n, %) | 18 (41%) | 9 (41%) | 9 (41%) | 1.00 |
|  |  |  |  |  |
| Total Gram-negative bacteria (n, %) | 7 (16%) | 4 (18%) | 3 (14%) | 0.68 |
| E. coli (n, %) | 3 (7%) | 2 (9%) | 1 (5%) | 0.55 |
| Klebsiella spp. (n, %) | 2 (5%) | 1 (5%) | 1 (5%) | 1.00 |
| Other Gram-negative bacilli (n, %) | 2 (5%) | 1 (5%) | 1 (5%) | 1.00 |
|  |  |  |  |  |
| Gram-positive bacteria (n, %) | 13 (30%) | 6 (27%) | 7 (32%) | 0.74 |
| Enterococcus spp. (n, %) | 4 (9%) | 2 (9%) | 2 (9%) | 1.00 |
| Staphylococcus spp. (n, %) | 3 (7%) | 2 (9%) | 1 (5%) | 0.55 |
| Streptococcus spp. (n, %) | 5 (11%) | 2 (9%) | 3 (14%) | 0.65 |
| MRSA (n, %) | 1 (2%) | 1 (5%) | 0 (0%) | 0.31 |
| Other Gram-positive bacilli (n, %) | 1 (2%) | 0 (0%) | 1 (5%) | 0.31 |
|  |  |  |  |  |
| Fungi (n, %) | 1 (2%) | 1 (5%) | 0 (0%) | 0.31 |
| Candida spp. (n, %) | 1 (2%) | 1 (5%) | 0 (0%) | 0.31 |
